# Molecular Network Analysis and Effector Gene Prioritization of Endurance-Training-Influenced Modulation of Cardiac Aging

**DOI:** 10.3390/genes16070814

**Published:** 2025-07-11

**Authors:** Mingrui Wang, Samuhaer Azhati, Hangyu Chen, Yanyan Zhang, Lijun Shi

**Affiliations:** 1 Department of Exercise Physiology, Beijing Sport University, Beijing 100084, China; wmrbody@bsu.edu.cn (M.W.); chy123@bsu.edu.cn (H.C.); yanyanzhang@bsu.edu.cn (Y.Z.); 2 School of Education, Beijing Sport University, Beijing 100084, China; sam@bsu.edu.cn; 3 Laboratory of Sports Stress and Adaptation, General Administration of Sport of China, Beijing Sport University, Beijing 100084, China; 4 Key Laboratory of Physical Fitness and Exercise, Ministry of Education, Beijing Sport University, Beijing 100084, China

**Keywords:** genetics, cardiac aging, endurance training, SMPX

## Abstract

Background/Objectives: Cardiac aging involves the progressive structural and functional decline of the myocardium. Endurance training is a well-recognized non-pharmacological intervention that counteracts this decline, yet the molecular mechanisms driving exercise-induced cardiac rejuvenation remain inadequately elucidated. This study aimed to identify key effector genes and regulatory pathways by integrating human cardiac aging transcriptomic data with multi-omic exercise response datasets. Methods: A systems biology framework was developed to integrate age-downregulated genes (*n* = 243) from the GTEx human heart dataset and endurance-exercise-responsive genes (*n* = 634) from the MoTrPAC mouse dataset. Thirty-seven overlapping genes were identified and subjected to Enrichr for pathway enrichment, KEA3 for kinase analysis, and ChEA3 for transcription factor prediction. Candidate effector genes were ranked using ToppGene and ToppNet, with integrated prioritization via the FLAMES linear scoring algorithm. Results: Pathway enrichment revealed complementary patterns: aging-associated genes were enriched in mitochondrial dysfunction and sarcomere disassembly, while exercise-responsive genes were linked to protein synthesis and lipid metabolism. TTN, PDK family kinases, and EGFR emerged as major upstream regulators. NKX2-5, MYOG, and YBX3 were identified as shared transcription factors. SMPX ranked highest in integrated scoring, showing both functional relevance and network centrality, implying a pivotal role in mechano-metabolic coupling and cardiac stress adaptation. Conclusions: By integrating cardiac aging and exercise-responsive transcriptomes, 37 effector genes were identified as molecular bridges between aging decline and exercise-induced rejuvenation. Aging involved mitochondrial and sarcomeric deterioration, while exercise promoted metabolic and structural remodeling. SMPX ranked highest for its roles in mechano-metabolic coupling and redox balance, with X-inactivation escape suggesting sex-specific relevance. Other top genes (e.g., KLHL31, MYPN, RYR2) form a regulatory network supporting exercise-mediated cardiac protection, offering targets for future validation and therapy.

## 1. Introduction

Cardiac aging is a complex, multi-layered biological process characterized by progressive structural and functional decline, which increases the risk of cardiovascular diseases (CVDs) such as heart failure, arrhythmia, atherosclerosis, and hypertension [1]. With the global demographic shift toward an aging population [1,2], understanding the molecular mechanisms underlying cardiac aging has become critical for developing effective interventions to mitigate age-associated cardiac dysfunction.

Endurance exercise is a well-recognized non-pharmacological intervention [1,3] that has been shown to alleviate various aging-related cardiac phenotypes, including myocardial fibrosis [4], mitochondrial dysfunction [5], and reduced contractile reserve [6]. However, the specific molecular networks and effector genes involved in mediating the cardioprotective effects of endurance training remain incompletely characterized [7,8,9,10,11,12]. Despite extensive evidence supporting the exercise-induced modulation of cardiac aging, the underlying regulatory architecture—particularly the central axis and its upstream drivers—remains poorly delineated due to the multifaceted and interconnected nature of the pathways involved [12,13,14], which hinders the development of precise “exercise-mimetic” therapies. For instance, while endurance training is known to reshape skeletal muscle enhancer activity [15], modulate DNA methylation patterns [16], and influence immune cell differentiation [17], the specific gene targets of these epigenetic changes are not well defined. Multi-tissue integrative analyses have shown that only ~5% of exercise-responsive genes exhibit consistent regulation across tissues [8], further complicating the identification of central regulatory nodes. This knowledge gap presents a major barrier to the effective translation of exercise biology into targeted anti-aging therapies.

Although exercise is widely regarded as an effective intervention to delay cardiac aging [1], directly obtaining human cardiac tissue samples to study post-exercise gene expression changes remains ethically [18] and technically challenging [19]. For instance, the collection of human tissue samples, including cardiac tissue, requires robust informed consent frameworks. However, ethical and regulatory discrepancies across jurisdictions may limit the permissible scope of sample use, thus restricting the samples’ application in cardiovascular research [18]. Recent studies utilizing CAGE (Cap Analysis of Gene Expression) technology have revealed transcriptional differences between healthy and failing human hearts [19], yet these approaches still rely on invasive tissue acquisition and cannot be considered truly noninvasive. Moreover, requiring elderly individuals to adhere strictly to long-term exercise protocols—with predefined frequency, intensity, and duration—followed by cardiac tissue sampling for comparison with control groups is not only ethically unfeasible but also practically unrealistic. On the one hand, even noninvasive alternatives [20] (e.g., metabolic surrogates) or indirect assessments via tissue homogenates [21] may fail to accurately reflect the transcriptional state of cardiomyocytes. On the other hand, older adults may face risks such as falls during training [22,23], heterogeneity in recovery capacity [24,25], or poor compliance [26,27]. Even when such interventions are completed, insufficient exercise intensity or volume may limit their anti-aging effects [28,29,30].

To address these limitations, we adopted an alternative strategy by integrating two high-quality public datasets and conducting Venn-based intersection analysis, aiming to identify core effector genes relevant to the “aging + exercise” context from a cross-species perspective. This strategy was built upon the robust transcriptomic data of endurance-training-responsive rat hearts provided by the MoTrPAC (Molecular Transducers of Physical Activity Consortium) project, which has been widely applied in molecular exercise research [10]. Despite interspecies differences, rats and humans share high degrees of conservation in cardiac structure, metabolic pathways, transcriptional regulation, and gene enrichment profiles [31,32,33,34]. Rat models have been extensively used in biomedical research since the early 20th century and are considered reliable surrogates for human biological mechanisms. In recent years, multi-omics integration (e.g., transcriptomics, proteomics, metabolomics) has enabled the resolution of subtle interspecies differences. Tools such as iAnimal facilitate the integration of cross-species datasets, especially when analyzing conserved regulatory mechanisms based on human cardiac resources like GTEx [35]. Thus, under current ethical constraints, cross-species integrative transcriptomic analysis represents not only a reliable and necessary alternative but also a highly informative approach. Compared to surrogate samples such as serum, transcriptomic profiling based directly on the target tissue (i.e., the heart) provides greater biological and physiological relevance [36]. Therefore, our strategy enables the systematic identification of key effector genes and regulatory mechanisms underlying the endurance-exercise-mediated attenuation of cardiac aging, without compromising human safety. This approach lays a theoretical foundation for future human studies, clinical interventions, and the development of exercise-mimetic therapeutics.

Traditional approaches that rely on single-omics or isolated pathway analyses are insufficient to capture the complexity of molecular networks. In contrast, systems biology approaches and multi-omics integration can uncover dynamic molecular interconnections [37,38]. Recent advances in computational tools such as gene set enrichment analysis (e.g., GSEA) and protein–protein interaction (PPI) network modeling have enabled the more comprehensive identification of key biological regulators [39,40]. Platforms like GeneSetCart offer flexible operations (e.g., intersection, union, and subtraction) across multiple public databases (e.g., KEGG, Reactome, MSigDB), making them powerful tools for analyzing complex biological processes [41]. Similarly, the ToppGene Suite supports both functional-annotation-based enrichment analysis (via ToppGene) and network-topology-based gene prioritization (via ToppNet) [42]. These tools have been widely adopted in systems biology research, particularly in integrative omics studies and effector gene identification.

In this study, we utilized these tools to systematically integrate transcriptomic signatures associated with cardiac aging—specifically, differentially expressed genes downregulated in older versus younger hearts from the Genotype-Tissue Expression (GTEx) dataset (GTEx Heart 20–29 vs. 60–69 Down) [43]—with endurance-training-responsive gene modules derived from the MoTrPAC (Molecular Transducers of Physical Activity Consortium) study (T58-Heart Consensus) [10]. By identifying genes that are shared across both biological states and analyzing their enrichment and network characteristics, we aimed to uncover the molecular interactions and regulatory mechanisms through which endurance training may delay or reverse cardiac aging. Most importantly, using functional similarity scoring (ToppGene) and network centrality scoring (ToppNet), we implemented the FLAMES linear integration model to derive a composite ranking (FinalScore) [44], enabling the multi-dimensional prioritization of potential effector genes. This study offers a new systems-level perspective on the endurance-training-mediated modulation of cardiac aging and provides a foundation for identifying therapeutic targets with translational potential.

## 2. Materials and Methods

### 2.1. Data Acquisition and Preprocessing

The “GTEx Heart 20–29 vs. 60–69 Down” gene set (*n* = 243) was obtained from the Genotype-Tissue Expression (GTEx) project [43] and reflects age-associated transcriptional changes in human cardiac tissue. This dataset was generated by comparing the RNA sequencing profiles of heart samples from young (ages 20–29) and older adult (ages 60–69) donors. Differential expression analysis was conducted using established pipelines (e.g., limma and DESeq2) [45,46], applying stringent statistical thresholds (adjusted *p*-value < 0.05 and log_2_ fold change <−1), followed by Bonferroni correction (α = 0.05/200,000) to control for multiple testing. A total of 243 genes were identified as significantly downregulated with age, many of which are involved in mitochondrial energy metabolism (e.g., ATP synthesis), oxidative stress response (e.g., antioxidant enzymes), and cell cycle regulation (e.g., DNA repair factors), reflecting the molecular features of age-related cardiac decline. Tissues were obtained via rapid autopsy or surgical donation, with all samples undergoing pathological validation and RNA integrity assessment (RIN > 7), and preserved using PAXgene Tissue fixatives to ensure RNA stability. The dataset is publicly accessible via the GTEx Portal (https://gtexportal.org/home/, accessed on 21 April 2025) or the dbGaP platform (https://www.ncbi.nlm.nih.gov/projects/gap/cgi-bin/study.cgi?study_id=phs000424.v8.p2, accessed on 21 April 2025).

The “T58-Heart Consensus” gene set (*n* = 634) was derived from the MoTrPAC (Molecular Transducers of Physical Activity Consortium) study [10], which investigated molecular responses to endurance training in F344 rats. Animals underwent standardized treadmill exercise (5 days/week at 70–75% VO_2_max), and heart tissues were harvested following 8 weeks of intervention. The dataset integrates multi-omics profiles (transcriptomics, proteomics, and metabolomics) collected across multiple time points (e.g., weeks 1, 2, 4, and 8) using high-throughput RNA-seq (HiSeq platform, ~50 million reads/sample) and mass spectrometry for both targeted and untargeted metabolomics. Quality control measures included RNA integrity verification (RIN > 7) and PAXgene Tissue preservation. Consensus analysis was applied to identify genes and molecular features showing consistent regulation across time points and experimental conditions. Raw data are available through the MoTrPAC Data Hub (https://motrpac-data.org, accessed on 21 April 2025).

To ensure cross-platform compatibility, all gene identifiers were standardized using NCBI Entrez Gene nomenclature, minimizing ambiguities in gene symbol interpretation [47]. Entrez Gene IDs were then mapped to the GeneSetCart and ToppGene Suite platforms for subsequent gene set enrichment, network analysis, and prioritization procedures.

### 2.2. Gene Set Network and Enrichment Analysis

Gene set overlap and functional enrichment analysis were conducted using GeneSetCart (v1.2.0) [41], which enables the integrative enrichment and topological evaluation of standardized gene sets. The two input sets—“GTEx Heart 20–29 vs. 60–69 Down” and “T58-Heart Consensus”—were analyzed to support a systems-level exploration of shared and distinct functional mechanisms.

GeneSetCart first expands the biological context of input gene sets using coexpression data, the literature co-occurrence, and protein–protein interaction (PPI) networks to generate functional extensions. The overlap between gene sets was visualized using multiple approaches, including Venn diagrams, hierarchical clustering heatmaps based on Jaccard similarity coefficients, SuperVenn diagrams, and UpSet plots, to comprehensively represent both shared features and unique signatures.

Functional enrichment analysis was performed using Enrichr, drawing from the WikiPathways database [48] and the Gene Ontology (GO) biological process annotation [49], to identify key pathways and biological processes significantly associated with each gene set [50]. GeneSetCart applies a hypergeometric test to evaluate the statistical significance of enrichment, estimating the probability of observing k or more genes from the input list overlapping with a given pathway by chance [51]. The enrichment *p*-value is calculated using the following formula:

(1) $$p=1-\sum_{i=1}^{k-1}\frac{\left(\begin{array}{c} K\\ i \end{array})(\begin{array}{c} N-K\\ n-i \end{array}\right)}{\left(\begin{array}{c} N\\ n \end{array}\right)}$$

where N denotes the total number of genes in the genome, K represents the number of known genes associated with a specific biological pathway or functional module, n is the total number of genes in the input gene set, and k refers to the number of input genes that fall within the given pathway. A smaller *p*-value indicates a higher degree of enrichment of that pathway within the target gene set, implying stronger biological relevance. The enrichment results are ranked by −log_10_( p-value) to highlight the most statistically significant functional pathways.

GeneSetCart also integrates the external tools KEA3 [52] and ChEA3 [53] to perform kinase and transcription factor (TF) enrichment analyses on the input gene sets. Kinase enrichment analysis was conducted using the Kinase Enrichment Analysis 3 (KEA3) tool [52], which infers upstream kinase activity based on the integration of proteomic and phosphoproteomic data. Specifically, KEA3 evaluates the statistical overlap between the input protein list and known kinase substrate sets using Fisher’s exact test and computes enrichment probabilities based on the hypergeometric distribution. For each kinase, KEA3 generates a composite enrichment score including both the Mean Rank (average integer rank across all reference libraries) and the Best Rank (best scaled rank across libraries). During analysis, KEA3 maps the input gene set to its corresponding proteins and queries 11 curated public databases—including PTMsigDB, prePPI, mentha, MINT, STRING, ChengKSIN, HIPPIE, BioGRID, PhosDAll, ChengPPI, and STRING.bind—to extract kinase-related associations. The resulting *p*-values are corrected for multiple testing using the Benjamini–Hochberg procedure to control the false discovery rate (FDR). The final kinase enrichment output is generated via the MeanRank Score integration across all sources. Transcription factor enrichment analysis was performed using ChEA3 (ChIP-X Enrichment Analysis 3) [53], which leverages multiple data types, including transcription factor–gene coexpression, TF–target regulatory associations, and TF–gene co-occurrence in the literature, to improve prediction accuracy. Similar to KEA3, Fisher’s exact test is used to assess the significance of the overlap between the input gene list and TF target sets, and *p*-values are adjusted using the Benjamini–Hochberg correction to control the FDR. ChEA3 integrates rankings from six reference databases—Literature ChIP-seq, GTEx Coexpression, ReMap ChIP-seq, Enrichr Queries, ENCODE ChIP-seq, and ARCHS4 Coexpression—and applies the MeanRank Score method to generate a final list of enriched transcription factors.

### 2.3. Gene Prioritization and Linear Integration

The ToppGene Suite prioritizes candidate genes through two core modules—ToppGene and ToppNet—which evaluate gene relevance based on functional annotations and protein–protein interaction (PPI) networks, respectively [42].

The process begins with the construction of a training set and a test set. The training set consists of genes known to be associated with the target phenotype or disease. In this study, we selected 206 differentially expressed genes unique to either the “GTEx Heart 20–29 vs. 60–69 Down” or the “T58-Heart Consensus” datasets (i.e., the non-overlapping portion) to form the training set. Their functional annotations—including GO terms, signaling pathways, mouse phenotypes, and protein interactions—were used to construct a representative feature profile. The test set comprised 37 overlapping genes between the two datasets, which served as candidate genes for prioritization.

Within the ToppGene module, functional enrichment analysis is first performed on the training genes using the ToppFun tool to identify significantly enriched annotation features. These annotations are then used to build the functional feature profile. For categorical annotations (e.g., GO terms), fuzzy semantic similarity is used to measure the similarity between the candidate (test) genes and the training profile. For quantitative annotations (e.g., expression profiles), Pearson correlation coefficients are computed to assess the similarity in expression patterns.

Finally, similarity scores across 19 annotation categories are integrated using a statistical combination approach—the Fisher’s inverse chi-squared method [54]—to produce a composite functional similarity score. The formula is as follows:

(2) $$S_{combined}=1-P_{fisher},\;P_{fisher}=-2\sum_{i=1}^{n}ln(pi)\sim X^{2}(2n)$$

where pi represents the significance p-value for the i-th annotation category, and n denotes the total number of annotation types considered. A higher composite score indicates a higher prioritization of the gene. To minimize potential bias due to incomplete annotation coverage, p-value correction was performed via genome-wide random sampling.

In the ToppNet module, candidate genes are prioritized based on topological properties within a protein–protein interaction (PPI) network. High-confidence PPI data are first integrated from major databases such as BioGRID, BIND, and HPRD to construct a comprehensive global interaction network. Using the training genes (i.e., seed genes), the system extracts their direct neighbors or K-step neighborhoods from the network to define a biologically relevant and locally coherent candidate space for analysis.

At the algorithmic level, ToppNet incorporates several classical and refined network-topology-based scoring methods to quantify the influence and regulatory potential of candidate genes within the network. Among these, the PageRank with Priors algorithm extends the traditional PageRank approach by incorporating prior knowledge, assigning higher scores to nodes that are more strongly connected to the seed genes [55]. The core equation is expressed as follows:

(3) $$PR(u)=\frac{1-d}{N}+d\sum_{v\in B(u)}\frac{PR(v)}{L(v)}$$

where PR(u) denotes the PageRank score of node u, d is the damping factor (typically set to 0.85), B(u) represents the set of all nodes linking to u, L(v) is the number of outbound edges from node v, and N is the total number of nodes in the network.

In addition to PageRank with Priors, the system also incorporates the K-step Markov diffusion model to capture the local propagation capacity of candidate genes within the PPI network. This model simulates the probability of information spreading from seed nodes to surrounding nodes over k steps, enabling the detection of genes with strong local connectivity and influence. Furthermore, centrality measures—such as degree centrality and betweenness centrality—are employed to identify key regulatory nodes with potential topological importance [56,57].

Based on the FLAMES framework [44], a linear scaling and integration strategy was employed to combine the prioritization scores from the ToppGene and ToppNet modules into a single FinalScore, thereby improving the accuracy of effector gene identification. The integration formula is defined as follows:

(4) FinalScore=α∗Gene_scaled+(1−α)∗Net_scaled+β∗IsTopNet

where α = 0.5 indicates the equal weighting of functional annotation (ToppGene score) and network topology (ToppNet score); β = 0.5 represents a penalty factor applied to hub genes; and IsTopNet is a binary indicator variable denoting whether the gene ranks within the top 5% of ToppNet scores (assigned 1 if true, otherwise 0). Gene_scaled and Net_scaled represent the normalized scores from ToppGene and ToppNet, respectively. This weighted scoring strategy balances functional similarity and network structure while further emphasizing the biological relevance of key regulatory nodes within the network. All candidate genes were ranked in descending order of FinalScore to prioritize the most likely effectors.

## 3. Results

### 3.1. Gene Set Overlap and Enrichment Analysis

#### 3.1.1. Gene Set Overlap

Using two complementary data sources, we first selected transcriptomic data from the cardiac tissue of individuals aged 20–29 and 60–69 years in the GTEx (Genotype-Tissue Expression) project. Differential expression analysis identified 243 genes significantly downregulated in the older group, representing the molecular signatures of functional decline with aging, such as mitochondrial metabolic dysfunction and impaired myocardial contractility [43].

Next, we constructed the T58-Heart Consensus exercise-responsive gene set by integrating multi-omics data (transcriptomics, proteomics, etc.) from rat cardiac tissue following 8 weeks of endurance training. This consensus set included 634 genes consistently upregulated or modulated by chronic exercise, involving pathways such as mitochondrial energy conversion, stress response activation, and sarcomeric remodeling—key mechanisms underlying cardiac protection [10].

**Figure 1:**
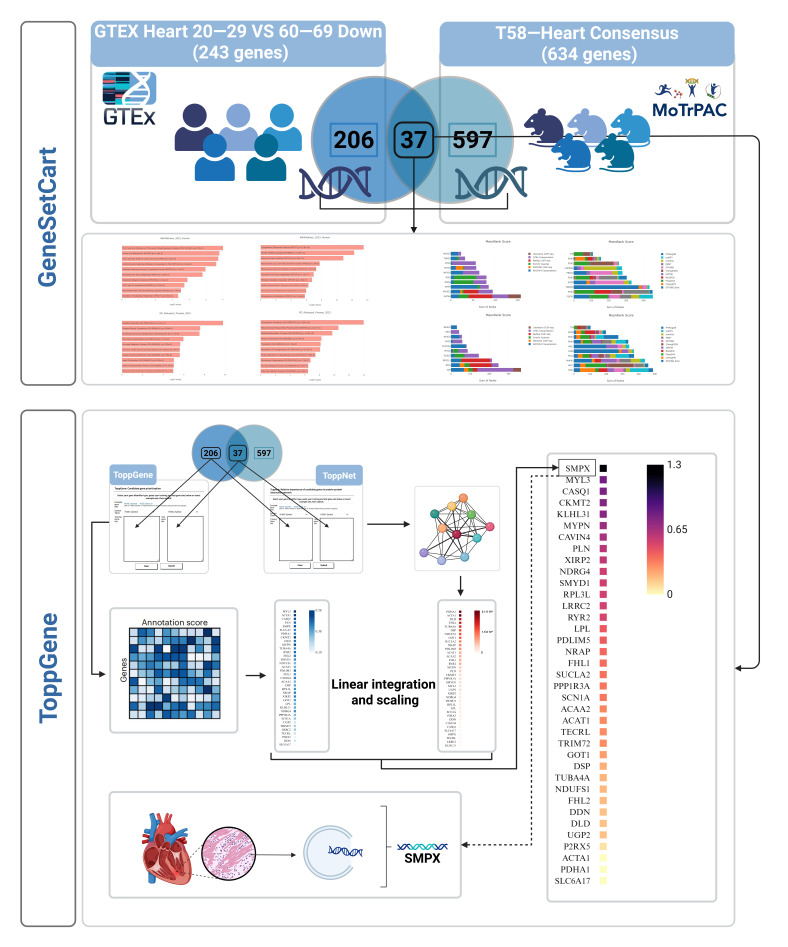
Overview of the research design. Differentially expressed genes related to cardiac aging were identified by integrating the GTEx Heart 20–29 vs. 60–69 Down and T58-Heart Consensus gene sets. Functional enrichment and network analyses were conducted using tools such as GeneSetCart and ToppGene. Priority scores from ToppGene and ToppNet were linearly integrated using the FLAMES framework to identify key molecular pathways and core regulatory genes. SMPX was ultimately selected as a potential effector gene. SMPX: Small Muscle Protein, X-Linked; MYL1: Myosin Light Chain 1; CASQ1: Calsequestrin 1; CKMT2: Creatine Kinase, Mitochondrial 2; KLHL31: Kelch Like Family Member 31; MYPN: Myopalladin; CAVIN4: Caveolae Associated Protein 4; PLN: Phospholamban; XIRP2: Xin Actin Binding Repeat Containing 2; NDRG4: N-Myc Downstream Regulated 4; SMYD1: SET and MYND Domain Containing 1; RPL3L: Ribosomal Protein L3-Like; LRRC2: Leucine Rich Repeat Containing 2; RYR2: Ryanodine Receptor 2; LPL: Lipoprotein Lipase; PDLIM5: PDZ and LIM Domain 5; NRAP: Nebulin Related Anchoring Protein; FHL1: Four and a Half LIM Domains 1; SUCNR1: Succinate Receptor 1; PPP1R3A: Protein Phosphatase 1 Regulatory Subunit 3A; SCN1A: Sodium Voltage-Gated Channel Alpha Subunit 1; ACAA2: Acetyl-CoA Acyltransferase 2; ACAT1: Acetyl-CoA Acetyltransferase 1; TECRL: Trans-2,3-Enoyl-CoA Reductase Like; TRIM72: Tripartite Motif Containing 72; G0S2: G0/G1 Switch 2; DDAH1: Dimethylarginine Dimethylaminohydrolase 1; TUBA4A: Tubulin Alpha 4a; NDUFS1: NADH:Ubiquinone Oxidoreductase Core Subunit S1; DDN: Dendrin; DLG2: Discs Large MAGUK Scaffold Protein 2; UGP2: UDP-Glucose Pyrophosphorylase 2; PRKX: Protein Kinase X-Linked; ACTA1: Actin Alpha 1, Skeletal Muscle; PDHA1: Pyruvate Dehydrogenase E1 Alpha 1 Subunit; SLC6A17: Solute Carrier Family 6 Member 17.

**Figure 2:**
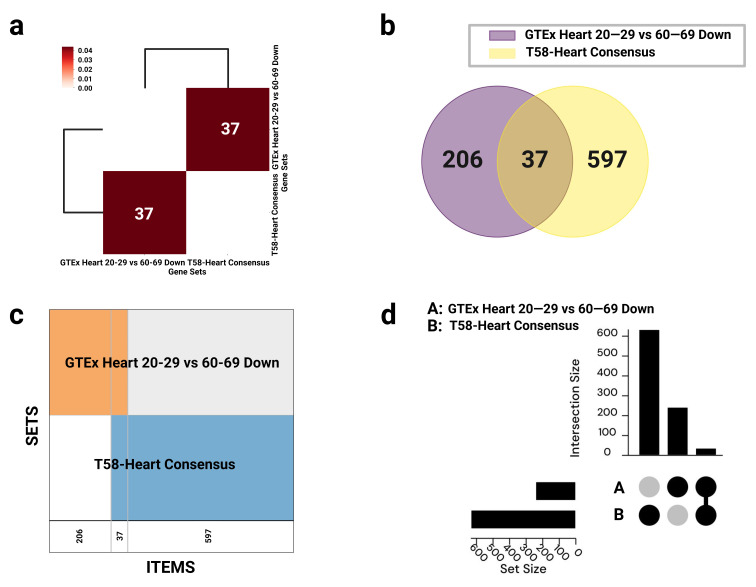
Visualization of gene set overlaps between GTEx Heart 20–29 vs. 60–69 Down and T58-Heart Consensus. (**a**) Hierarchical clustering heatmap based on Jaccard similarity scores, with annotations indicating the number of overlapping genes; interactive version available via GeneSetCart: https://genesetcart.cfde.cloud/visualize/cma3yx2om00yw21dql4252u5d?checked=0,1&type=Heatmap. (**b**) Venn diagram illustrating the intersection between the two gene sets; interactive version: https://genesetcart.cfde.cloud/visualize/cma3yx2om00yw21dql4252u5d?checked=0,1&type=Venn. (**c**) SuperVenn diagram displaying regions of overlapping genes, with counts shown at the bottom of each segment; interactive version: https://genesetcart.cfde.cloud/visualize/cma3yx2om00yw21dql4252u5d?checked=0,1&type=SuperVenn. (**d**) UpSet plot representing the distribution and intersection of gene sets A (GTEx) and B (T58-Heart); interactive version: https://genesetcart.cfde.cloud/visualize/cma3yx2om00yw21dql4252u5d?checked=0,1&type=UpSet. SETS: gene sets; ITEMS: gene items; set size: the total number of genes in each individual dataset; intersection size: the number of shared or uniquely present genes in each set combination.

An intersection analysis identified 37 overlapping genes between the two datasets. These genes were downregulated with aging yet significantly upregulated or activated in response to endurance training, suggesting they may function as key regulatory nodes in the reversal of aging-related cardiac phenotypes through exercise (Figure 1 and Figure 2).

#### 3.1.2. Functional Enrichment Analysis

**Figure 3:**
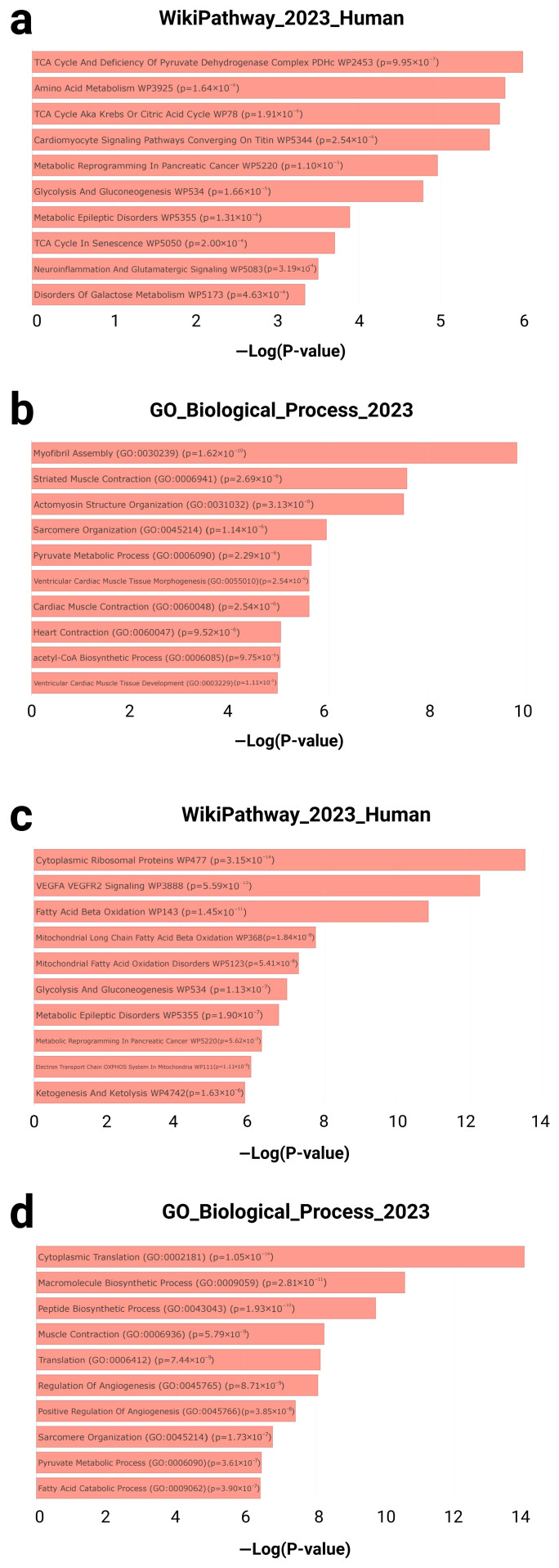
Enrichment analysis results for gene sets associated with cardiac aging and exercise response. (**a**) Top 5 enriched pathways from the WikiPathway_2023_Human library and (**b**) top 5 enriched biological processes from the GO biological processes library for the GTEx Heart 20–29 vs. 60–69 Down gene set. Results available at Enrichr: https://maayanlab.cloud/Enrichr/enrich?dataset=7b89832d110d853d2be939c498d86a62. (**c**) Top 5 enriched pathways and (**d**) biological processes for the T58-Heart Consensus gene set, with full results accessible via Enrichr: https://maayanlab.cloud/Enrichr/enrich?dataset=1e277c793270336a30ef9a59a6ee1dc7. WikiPathway_2023_Human: An open-access database of human molecular pathways used to identify metabolic, signaling, and disease-related pathways associated with specific gene sets. The database contains a large number of manually curated biological pathways and is commonly used in functional enrichment analyses to explore potential regulatory mechanisms. GO_Biological_Process_2023: a sub-ontology within the Gene Ontology database, used to characterize genes based on their roles in higher-level biological processes. −Log(*p*-value): The negative base-10 logarithm of the enrichment *p*-value, used to represent the statistical significance of each enriched term. Higher values indicate greater significance.

Two manually curated gene sets were subjected to enrichment analysis: the GTEx heart downregulated gene set (20–29 vs. 60–69 years; *n* = 243) and the T58-Heart Consensus exercise-responsive gene set (*n* = 634) (Figure 3). Both sets were independently analyzed using the Enrichr platform to identify statistically significant pathway and biological process enrichment.

In the GTEx downregulated gene set, multiple pathways related to cardiac metabolism and contractile structure were significantly enriched. According to WikiPathways, top-ranked terms included the TCA cycle and deficiency of the pyruvate dehydrogenase complex (*p* = 9.95 × 10^−7^), amino acid metabolism (*p* = 1.64 × 10^−6^), and cardiomyocyte signaling pathways converging on titin (*p* = 2.54 × 10^−6^) (Figure 3a). Concurrently, Gene Ontology (GO) biological process analysis identified highly enriched terms such as myofibril assembly (GO: 0030239; *p* = 1.62 × 10^−10^), striated muscle contraction (GO: 0006941; *p* = 2.69 × 10^−8^), and pyruvate metabolic process (GO: 0006090; *p* = 2.29 × 10^−6^) (Figure 3b).

In contrast, the T58-Heart Consensus gene set exhibited an enrichment pattern centered on mitochondrial energy metabolism and protein synthesis. Top WikiPathways terms included cytoplasmic ribosomal proteins (*p* = 3.15 × 10^−14^), the VEGFA–VEGFR2 signaling pathway (*p* = 5.59 × 10^−13^), and fatty acid beta-oxidation (*p* = 1.45 × 10^−11^) (Figure 3c). GO biological process enrichment further revealed significant terms such as cytoplasmic translation (GO: 0002181; *p* = 1.05 × 10^−14^), muscle contraction (GO: 0006936; *p* = 5.79 × 10^−9^), and fatty acid catabolic process (GO: 0009062; *p* = 3.90 × 10^−7^) (Figure 3d).

Taken together, the genes downregulated with cardiac aging were primarily associated with diminished contractile capacity and metabolic activity, while the endurance-training-responsive genes were significantly enriched in pathways related to mitochondrial function and protein biosynthesis. These findings suggest a compensatory cardiac remodeling mechanism induced by exercise.

#### 3.1.3. Kinase Enrichment Analysis

**Figure 4:**
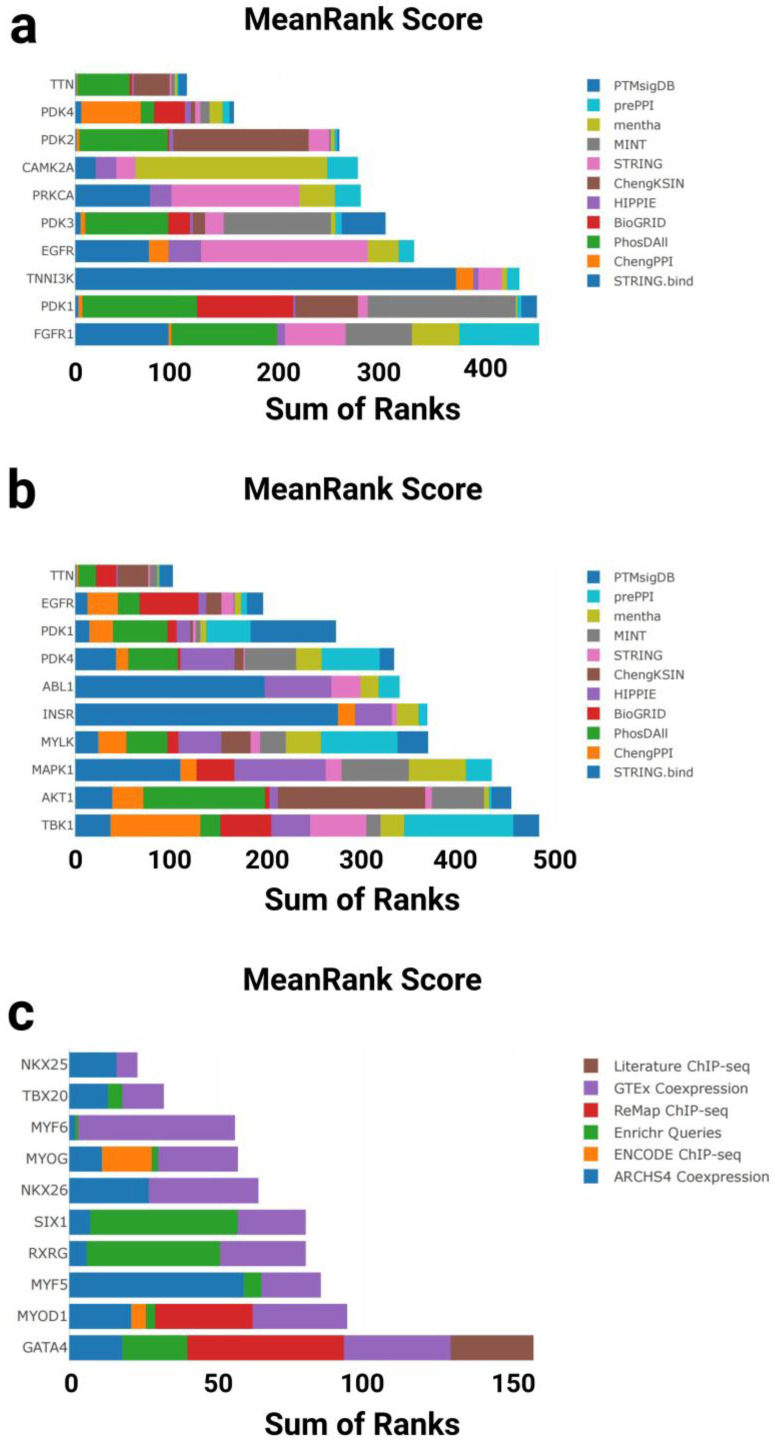

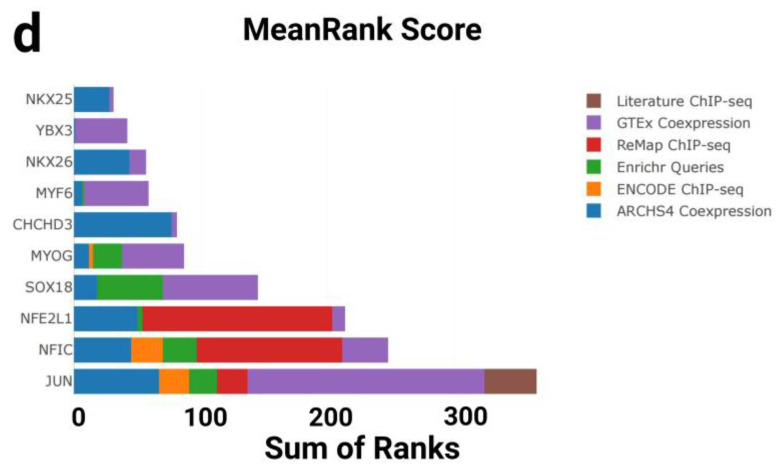
Kinase and transcription factor enrichment analysis of gene sets associated with cardiac aging and exercise response. (**a**) Top 10 enriched kinases for the GTEx Heart 20–29 vs. 60–69 Down gene set based on mean rankings from KEA3. (**b**) Top 10 enriched kinases for the T58-Heart Consensus gene set. (**c**) Top 10 enriched transcription factors (TFs) for the GTEx Heart 20–29 vs. 60–69 Down gene set based on mean rankings from ChEA3. (**d**) Top 10 enriched TFs for the T58-Heart Consensus gene set. PTMsigDB: Post-Translational Modification Signature Database; prePPIs: Predicted Protein–Protein Interactions; mentha: MEtabolic Network THrough Averaging; MINT: Molecular INTeraction Database; STRING: Search Tool for the Retrieval of Interacting Genes/Proteins; ChengKSIN: Cheng Kinase–Substrate Interaction Network; HIPPIE: Human Integrated Protein–Protein Interaction rEference; BioGRID: Biological General Repository for Interaction Datasets; PhosDAll: Phosphorylation Data Aggregated from All Resources; ChengPPI: Cheng Protein–Protein Interaction Dataset; STRING.bind: STRING Binding Evidence Subset; TTN: titin; PDK1: Pyruvate Dehydrogenase Kinase Isozyme 1; PDK2: Pyruvate Dehydrogenase Kinase Isozyme 2; PDK3: Pyruvate Dehydrogenase Kinase Isozyme 3; PDK4: Pyruvate Dehydrogenase Kinase Isozyme 4; CAMK2A: Calcium/Calmodulin-Dependent Protein Kinase II Alpha; PRKCA: Protein Kinase C Alpha; EGFR: Epidermal Growth Factor Receptor; FGFR1: Fibroblast Growth Factor Receptor 1; TNNI3K: Troponin I-Interacting Kinase; ABL1: ABL Proto-Oncogene 1; INSR: Insulin Receptor; MYLK: Myosin Light Chain Kinase; MAPK1: Mitogen-Activated Protein Kinase 1; AKT1: AKT Serine/Threonine Kinase 1; TBK1: TANK-Binding Kinase 1; Literature ChIP-seq: transcription factor–target gene associations curated from published chromatin immunoprecipitation (ChIP)-sequencing studies; GTEx Coexpression: TF rankings based on gene expression correlations in GTEx human tissue datasets; ReMap ChIP-seq: high-confidence TF–DNA interactions derived from ReMap ChIP-seq database; Enrichr Queries: co-enrichment analysis of TF targets from user-submitted gene sets in the Enrichr platform; ENCODE ChIP-seq: TF binding data from the ENCODE consortium’s standardized ChIP-seq experiments; ARCHS4 Coexpression: TF–gene association based on large-scale RNA-seq coexpression data from ARCHS4; NKX2-5: NK2 Homeobox 5; TBX20: T-box Transcription Factor 20; MYF6: Myogenic Factor 6; MYOG: Myogenin; NKKX2-6: NK2 Homeobox 6; SIX1: SIX Homeobox 1; RXRG: Retinoid X Receptor Gamma; MYF5: Myogenic Factor 5; MYOD1: Myogenic Differentiation 1; GATA4: GATA Binding Protein 4; YBX3: Y-box Binding Protein 3; CHCHD3: Coiled-Coil-Helix-Coiled-Coil-Helix Domain Containing 3; SOX18: SRY-Box Transcription Factor 18; NFE2L1: Nuclear Factor, Erythroid 2 Like 1; NFIC: Nuclear Factor I C-type; JUN: Jun Proto-Oncogene, AP-1 Transcription Factor Subunit.

To explore potential upstream regulatory mechanisms, KEA3 (Kinase Enrichment Analysis 3) was applied separately to the two gene sets to identify kinases potentially responsible for the observed gene expression changes (Figure 4a,b). KEA3 ranks kinases based on their average position across multiple protein–protein interaction (PPI) databases, highlighting those most enriched within each gene set.

In the GTEx downregulated gene set, the top-enriched kinases were primarily associated with structural and metabolic regulation in the heart. Notably, PDK1, EGFR, PDK3, PRKCA, and TNNI3K ranked among the top hits, with PDK1 and PDK3 showing the highest enrichment—both key regulators of glucose metabolism. The calcium/calmodulin-dependent kinase CAMK2A, which regulates excitation–contraction coupling in cardiomyocytes, also showed significant enrichment. In addition, TTN, a giant sarcomeric protein linked to myocardial elasticity and aging, was detected among the top-ranked hits (Figure 4a).

In contrast, the T58-Heart Consensus gene set exhibited a distinct kinase enrichment profile. Leading kinases included AKT1, MAPK1, MYLK, INSR, and ABL1, all of which are implicated in stress response and cardiac hypertrophy, particularly under physiological conditions. PDK4 and PDK1 were again enriched, reinforcing the importance of the pyruvate dehydrogenase regulatory axis in exercise-induced cardiac adaptation. EGFR and TTN were also enriched in this dataset, suggesting a partially shared regulatory network between aging and endurance training (Figure 4b).

Collectively, these findings indicate that cardiac aging and endurance exercise engage both overlapping and distinct kinase signaling axes. Of particular interest are metabolic kinases (e.g., the PDK family), mechanosensitive regulators (e.g., TNNI3K and MYLK), and key pathways involved in calcium signaling and structural remodeling. These results provide new insight into the molecular adaptation of the heart under aging and exercise conditions.

#### 3.1.4. Transcription Factor Enrichment Analysis

To further investigate upstream regulatory mechanisms at the transcriptional level, we performed transcription factor (TF) enrichment analysis on both gene sets using the ChEA3 tool (Figure 4c,d). This analysis integrates multiple data sources to identify and rank transcription factors most strongly associated with the input gene sets.

In the GTEx downregulated gene set, ChEA3 identified a set of key transcription factors associated with cardiac development. Top-ranked TFs included GATA4, MYOD1, MYF5, MYOG, MYF6, and NKX2-5, all of which play central roles in cardiac morphogenesis and muscle differentiation. Notably, TBX20 and NKX2-6, which are involved in early heart field patterning and chamber specification, also showed strong enrichment. These findings suggest that the transcriptional downregulation observed in aging hearts may reflect the reduced activity of developmental regulatory programs (Figure 4c).

In contrast, the T58-Heart Consensus gene set (representing the endurance training response) exhibited a partially overlapping but distinct enrichment profile. Highly ranked TFs included JUN, NFIC, NFE2L1, SOX18, and YBX3, which are involved in stress response, metabolic adaptation, and angiogenic regulation. Interestingly, MYOG, MYF6, and NKX2-5 again ranked highly in this dataset, further supporting the hypothesis that these core transcription factors may be reactivated or functionally remodeled during exercise-induced cardiac remodeling (Figure 4d).

Taken together, ChEA3 analysis revealed both shared and divergent transcriptional regulatory patterns between aging and exercise. While both conditions involve key factors such as MYOG and NKX2-5, their regulatory direction and network context differ significantly: aging appears to suppress transcriptional programs linked to development and contractility, whereas endurance training may restore or reorganize these networks to promote cardiac plasticity and functional adaptation.

#### 3.1.5. Effector Gene Prioritization

To prioritize candidate effector genes potentially mediating the anti-aging effects of endurance training on the heart, we developed an integrative ranking framework combining functional similarity scoring (ToppGene), network centrality metrics (ToppNet), and a linear weighted model (FLAMES). Among the 37 intersecting genes identified from the overlap between the GTEx aging-downregulated and T58-Heart Consensus training-responsive gene sets, SMPX (Small Muscle Protein, X-linked) ranked highest based on the final weighted score (FinalScore = 1.307) (Figure 1, Table A1).

Although MYL3 (Myosin Light Chain 3) received the top rank in functional annotation similarity, its relatively low centrality in the protein–protein interaction (PPI) network resulted in a slightly lower overall score compared to SMPX (see Figure 1, Table A1 ). As a structural sarcomeric protein, MYL3 plays well-documented roles in muscle contraction and cardiomyopathies, but its limited topological influence in the interaction network reduced its composite ranking.

In contrast, SMPX demonstrated both strong functional enrichment and high network centrality, underscoring its dual role in mechanosensation and stress-adaptive signaling. Previous studies have shown that SMPX stabilizes membrane–cytoskeleton junctions and responds to mechanical load, potentially regulating ACTN2 (α-actinin-2) and calcium signaling pathways. However, the integrative prioritization framework supports SMPX as the most promising effector gene. Due to its intersecting roles in metabolic regulation, mechanical stability, and stress signaling, SMPX may serve as a key node in exercise-induced cardiac rejuvenation. These findings highlight the practical utility of combining pathway-level, annotation-based, and network-based features to identify genes with both mechanistic relevance and translational potential.

## 4. Discussion

Although transcriptomic and proteomic studies have proposed numerous candidate genes associated with exercise-induced cardiac remodeling, few studies have systematically integrated multi-dimensional aging datasets with network-level information to prioritize key effector genes [58,59,60,61,62,63,64,65]. In this study, we constructed an integrative analytical framework by leveraging cross-species omics data from the GTEx and MoTrPAC projects, combining pathway-based pre-filtering with network topological ranking to identify putative effectors involved in the protective effects of endurance training against cardiac aging. Unlike traditional research that focuses on canonical regulators such as PGC-1α, SIRT1, or AMPK [66,67,68,69,70], this approach moves beyond a candidate-gene-driven paradigm by emphasizing the emergent properties of molecular networks, thereby enhancing both the system-level insight and translational potential of effector gene selection.

### 4.1. Functional Enrichment Analysis

The functional enrichment analysis revealed that cardiac aging and endurance training involve distinct but complementary molecular regulatory mechanisms, primarily centered around energy metabolism homeostasis and sarcomeric structural integrity.

In the GTEx aging-associated gene set [43], there was significant downregulation of the tricarboxylic acid (TCA) cycle and the pyruvate dehydrogenase complex (PDHc) (WP2453, *p* = 9.95 × 10^−7^), consistent with prior findings suggesting impaired pyruvate metabolism and mitochondrial energy crisis in aged myocardium [71]. This metabolic decline is closely linked to the reduced expression of key genes such as PDHA1 [72,73]. Furthermore, the enrichment of terms like myofibril assembly (GO: 0030299; *p* = 1.62 × 10^−10^) and cardiac muscle contraction (GO: 0060048; *p* = 2.54 × 10^−6^) indicates deterioration in contractile function, which has been previously associated with sarcomere disorganization and impaired force generation [74].

Mitochondrial dysfunction during aging often leads to reduced oxidative phosphorylation capacity, the accumulation of reactive oxygen species (ROS), and a decline in fatty acid oxidation efficiency. These defects may trigger the compensatory enhancement of glycolysis, as reflected by the shared enrichment of glycolysis/gluconeogenesis pathways in both the GTEx and T58 datasets—potentially driving a maladaptive cycle of metabolic inflexibility [75,76,77].

In contrast, enrichment analysis of the T58-Heart Consensus gene set suggests that endurance training may reverse these aging-related changes through metabolic network remodeling. Key findings include the upregulation of fatty acid β-oxidation (WP143, *p* = 1.45 × 10^−11^) [78,79] and activation of the mitochondrial oxidative phosphorylation system (WP111, *p* = 1.11 × 10^−6^) [80,81], with regulators such as CPT1B supporting more efficient lipid-based energy supply [82,83]. Additionally, genes like NDUFS1, encoding subunits of mitochondrial complex I, may enhance electron transport chain (ETC) function [84,85]. The observed upregulation of cytoplasmic translation (GO: 0002181; *p* = 1.05 × 10^−14^) may also promote ribosome biogenesis and protein turnover [86,87,88], further contributing to a “metabolic–mechanical” dual-protection strategy against cardiac aging.

### 4.2. Kinase Enrichment Analysis

TTN (titin) emerged as the top-ranked kinase-associated gene in both the GTEx and T58 datasets based on the MeanRank Score, suggesting a central role in the structural and functional remodeling of the aging heart. This finding is supported by mechanistic studies. For instance, Z-disk-anchored titin deletion models in mice have demonstrated that the loss of titin leads to sarcomeric disintegration, Z-disk disorganization, and the activation of mechanoresponsive proteins. These changes result in increased muscle stiffness and cardiac dysfunction, closely recapitulating the muscle remodeling observed in patients with severe myosinopathies [89]. Moreover, the A168–M1 region of titin has been identified as a mechanosensitive kinase platform that forms a ubiquitin-dependent regulatory complex with proteins such as MuRF1 and Nbr1/p62, responding to mechanical stress and modulating sarcomeric autophagic degradation [90]. These findings suggest that titin not only plays a structural role in sarcomere maintenance but also acts as a signaling hub, coupling mechanical stimuli to proteostasis regulation. The observed TTN enrichment and inferred kinase activity in the aging heart may thus reflect the activation of these dual functions, reinforcing TTN’s potential as a therapeutic target for cardiac aging.

In parallel, the PDK family (PDK1/2/3/4) may contribute to metabolic dysfunction during aging by phosphorylating and inhibiting pyruvate dehydrogenase complex (PDHc) activity, thereby shifting cardiac metabolism toward glycolysis and exacerbating mitochondrial energy deficits [91,92,93]. CAMK2A, ranked fourth by the MeanRank Score, may promote arrhythmogenic risk in the aging myocardium through abnormal activation and interference with RyR2 channel function, disrupting calcium homeostasis [94,95].

In the exercise-responsive T58 dataset, EGFR was the second most enriched kinase, suggesting a potential regulatory role in cardiovascular adaptation to endurance training. Previous studies have shown that EGFR signaling participates in angiotensin II (AT1R)-mediated vascular remodeling and inflammation, with the downstream activation of the ERK, p38MAPK, and STAT pathways influencing vascular structure and function [96]. EGFR also plays a critical role in bone marrow-derived cells; its deletion in these cells leads to cardiac hypertrophy, reduced capillary density, and impaired myocardial repair, indicating its importance in cardiac homeostasis and stress response [97]. Furthermore, enhanced VEGFB signaling has been shown to reverse diastolic dysfunction and neurodegeneration in the aging heart, and EGFR, as a key node in the VEGF signaling network, may mediate cardiovascular neurotrophic coupling via pathways such as STAT3 [97].

Another notable kinase, ABL1, may also contribute to exercise-induced cardiac adaptation and stress regulation. ABL1 interacts with factors like p53 to modulate cardiomyocyte responses to external insults, such as doxorubicin-induced mitochondrial toxicity and cardiomyopathy, involving pathways related to mitochondrial homeostasis, autophagy, apoptosis, and DNA damage response [98]. Additionally, ABL1 kinase activity is closely linked to cardiac development and structural integrity. Germline mutations in ABL1 have been associated with congenital heart defects, skeletal abnormalities, and developmental syndromes, often accompanied by elevated kinase activity [99]. These findings suggest that ABL1 is both a key component of the exercise-induced stress-sensing network and a modulator of cardiomyocyte adaptation under mechanical and metabolic reprogramming.

Collectively, these kinases integrate mechanical stress, metabolic feedback, and inflammatory signaling to form a dynamic regulatory network governing both cardiac aging and exercise-induced adaptation.

### 4.3. Transcription Factor Enrichment Analysis

Transcription factor (TF) enrichment analysis revealed that the regulatory network of cardiac aging is prominently centered around NKX2-5, which ranked first in both the GTEx aging-related gene set and the T58 exercise-responsive gene set. This suggests a high degree of transcriptional overlap between cardiac aging and exercise-induced cardiac remodeling. Previous studies have shown that exercise training—especially high-intensity exercise—not only improves post-infarction cardiac function but also upregulates TFs associated with cardiac development such as NKX2-5, GATA4, and CITED4 while simultaneously activating cardiac regeneration markers including c-Kit and Sca-1, indicating a transcriptional regulatory role for exercise in myocardial repair [100].

Furthermore, NKX2-5 has been identified as a key regulator of cardiac regeneration, as its deletion results in impaired regenerative capacity and dysregulated injury response. Mechanistically, NKX2-5 is involved in the activation of proteolysis, mitochondrial metabolism, and cell cycle re-entry [101]. Thus, the strong enrichment of NKX2-5 observed in the aging heart may reflect the reactivation of a regeneration-associated transcriptional network triggered by endurance training, supporting its role as a central hub in reversing cardiac aging.

TBX20, ranked second in the GTEx dataset, also plays critical biological roles. On the one hand, TBX20 is essential in the direct cardiac reprogramming of human fibroblasts, where it cooperates with cardiac enhancers to activate myocardial gene expression, enhancing calcium transients, mitochondrial respiration, and contractile function—thereby promoting cardiomyocyte maturation and performance [102]. On the other hand, developmental studies show that TBX20 regulates extracellular matrix genes (e.g., Vcan) and cell migration in endocardial cells, contributing to atrioventricular and outflow tract septation, while its ability to bind distant enhancers underscores its epigenomic regulatory impact [103].

MYF6 and MYOG, ranked third and fourth in the GTEx aging gene set, respectively, are classically associated with skeletal muscle development but may also play roles in cardiac aging. MYF6 has been shown to maintain muscle stem cell homeostasis and regenerative capacity through the regulation of secreted factors (e.g., EGF) in myofibers. MYF6 deficiency leads to stem cell depletion and loss of differentiation control, highlighting its role in stem cell niche maintenance and anti-aging signaling [104].

Meanwhile, Myogenin (MYOG) is significantly upregulated in aged myogenic progenitors and coexpressed with CD74, marking a senescent subpopulation with reduced regenerative potential. Recent findings implicate miR-501 and its target Esrrg in modulating the MYOG/CD74 axis, linking its dysfunction to impaired sarcomere formation and reduced tissue elasticity [105]. Together with our data, these findings suggest that MYF6 and MYOG, although primarily studied in skeletal muscle, may contribute to stem cell dysfunction, sarcomere instability, and impaired tissue repair in cardiac aging, indicating a potential cross-tissue role in aging regulation.

Additionally, YBX3 ranked second in the T58 exercise-responsive gene set, suggesting its potential involvement in exercise-induced adaptation. Traditionally considered an RNA-binding protein, YBX3 is increasingly recognized for its role in metabolic regulation and tissue adaptation. It has been identified as a key thermogenic factor induced by cold exposure and β-adrenergic signaling. In brown adipocytes, YBX3 stabilizes Slc3a2 and Pparg mRNAs, enhancing branched-chain amino acid metabolism, mitochondrial oxidative phosphorylation, and thermogenesis [106]. In vivo, YBX3 overexpression improves diet-induced metabolic dysfunction, while its knockdown suppresses thermogenesis and exacerbates obesity phenotypes.

Given the high energy demands and metabolic flexibility required during endurance training, the observed enrichment of YBX3 in the T58 dataset may reflect its role in coordinating exercise-induced metabolic remodeling and mitochondrial optimization, particularly across skeletal muscle and adipose tissue [107,108]. These findings underscore the need for further investigation into YBX3’s mechanistic role in exercise adaptation.

### 4.4. Effector Gene Prioritization

By applying the FLAMES linear scaling integration strategy to functional similarity (ToppGene) and network centrality (ToppNet) scores, we prioritized candidate effector genes from the 37 overlapping genes. Although MYL3 ranked first in functional annotation similarity, reflecting its well-established role in sarcomere assembly and myocardial contractility, it was slightly outperformed by SMPX in the final composite score due to the weighting of network centrality. As a classical structural protein, MYL3 is functionally specific but relatively limited in terms of system-level connectivity and regulatory potential [109,110]. Studies have shown that MYL3 can interact with circRNA (circ-0001283) to regulate protein stability and activate PI3K/Akt/mTOR- and ERK-mediated autophagy signaling, thereby promoting cardiac hypertrophy progression [109]. Loss-of-function mutations in MYL3 have been implicated in recessive cardiomyopathy and sudden cardiac death, highlighting its critical role in the contractile machinery [110]. However, Ma et al. recently demonstrated in iPSC-derived cardiomyocyte models carrying the MYL3 c.170C>A variant that this mutation failed to elicit classic hypertrophic cardiomyopathy (HCM) phenotypes, suggesting it may be a benign variant [111]. This finding challenges earlier pathogenic predictions and introduces uncertainty regarding MYL3’s classification as a high-confidence effector gene.

Calsequestrin 1 (CASQ1), a sarcoplasmic reticulum calcium-binding protein, ranked just below MYL3 and plays a vital role in calcium release regulation and rhythm stabilization. Beyond its well-established buffering capacity, CASQ1 has also been shown to function as a dynamic Ca^2+^ sensor, a structural organizer of terminal cisternae, and a modulator of extracellular calcium entry—all of which are critical to maintaining skeletal and cardiac excitation–contraction coupling fidelity [112]. CASQ1 deficiency has been linked to malignant hyperthermia (MH) and exercise heat stroke (EHS)-like responses [113]. Notably, endurance training has been shown to mitigate the lethal risk in CASQ1-deficient models by improving mitochondrial function and reducing oxidative stress [114]. CKMT2, another top candidate, is downregulated in diabetic hearts and is associated with mitochondrial dysfunction; exercise can restore its expression and enhance oxidative phosphorylation, suggesting its potential role as a mitochondrial homeostasis modulator [115].

Beyond CKMT2, several other candidate genes prioritized based on the FinalScore ranking (positions 4–37) also warrant in-depth discussion, as they play indispensable roles in sarcomeric integrity, calcium homeostasis, metabolic remodeling, and stress adaptation. KLHL31 and MYPN are two key structural regulators that maintain sarcomere organization and transduce mechanical signals. KLHL31 knockout in mice leads to sarcoplasmic reticulum (SR) dilation and Z-disc disorganization, likely due to the impaired degradation of Filamin-C, highlighting KLHL31’s pivotal role in SR–sarcomere coupling [116]. MYPN, through its interaction with MRTF-A and regulation of actin bundling, enhances SRF-dependent transcriptional programs and preserves Z-line structure under mechanical stress. Knockout models exhibit myofiber atrophy and exercise-induced damage [117]. CAVIN4, a caveolae-associated structural protein, is critical for T-tubule maturation and excitation–contraction coupling. Its deletion disrupts membrane remodeling and impairs calcium transients in response to mechanical stimulation, indicating its essential function in endurance-adapted myocardium [118]. Similarly, PLN and RYR2, two canonical regulators of SR calcium homeostasis, were prioritized for their roles in cardiomyocyte development and rhythmic control. The pentamer-to-monomer transition of PLN is thought to be a regulatory switch for SERCA inhibition [119], while the loss of RYR2 induces ER stress and suppresses protein synthesis through ATF4 activation, linking calcium flux to cardiomyocyte maturation [120].

In terms of structural regulation, genes such as XIRP2, PDLIM5, and FHL1/FHL2 further reveal the crosstalk between mechanical signaling and transcriptional control in hypertrophic remodeling. XIRP2, regulated by MEF2A in the angiotensin II signaling pathway, is involved in fibrosis and apoptosis [121]; PDLIM5 is spliced via RBPMS, contributing to sarcomeric protein diversity and contractile precision [122]; and FHL1/FHL2 interact with cytoskeletal and nuclear signaling pathways to regulate contractility and autophagy, with mutations closely associated with hypertrophic cardiomyopathy and related myopathies [123,124].

In addition, several candidate genes reveal novel metabolic or translational regulatory axes. SMYD1, a muscle-specific histone methyltransferase, activates PGC-1α-mediated mitochondrial transcription and respiration, and its knockout results in early energetic failure of the heart [125]; RPL3L-based cardiac-specific ribosomes regulate the elongation and translation of contraction-related proteins [126]; and LRRC2, a downstream target of PGC-1α, promotes mitochondrial remodeling in hypertrophic environments [127]. NDUFS1, a core subunit of mitochondrial complex I, is downregulated in hypertrophic myocardium, and its suppression increases ROS production and mitochondrial fragmentation [74].

Other functionally diverse candidates further enrich the landscape of cardiac remodeling. TRIM72 (MG53) mediates membrane repair under oxidative stress through disulfide-linked oligomerization and lipid binding [128]; GOT1 maintains aspartate production under OXPHOS inhibition, supporting TCA cycle anaplerosis and stress resilience [129]. While DDN is primarily expressed in the nervous system, its role in the Kibra-dependent signaling pathway suggests potential involvement in heart–brain metabolic crosstalk [130]. Similarly, NDRG4, a heart-specific member of the NDRG family, has been linked to stress response and cardiac homeostasis. Its downregulation in diseased tissue and differential splicing between the heart and brain imply cardioprotective roles during aging and stress adaptation [131].

In the context of electrophysiological regulation and arrhythmia risk, several candidates also deserve attention. TECRL mutations impair SR calcium loading and prolong action potential repolarization, presenting a mixed phenotype of LQTS and CPVT [132]; PPP1R3A, a regulatory subunit of PP1, anchors to RyR2 and PLN, modulating their phosphorylation state and protecting against aberrant calcium release and atrial structural instability [133]; SCN1A mutations increase sodium current and spontaneous beating in iPSC-derived cardiomyocytes, potentially linking epilepsy-associated genes to cardiac excitability [134].

Moreover, several emerging metabolic regulators broaden our understanding of cardiac aging mechanisms. ACAA2 and ACAT1 participate in fatty acid β-oxidation and cholesterol esterification, respectively; their dysregulation is associated with inflammatory activation and lipid imbalance in cardiometabolic diseases [135,136]. LPL, a key enzyme for triglyceride hydrolysis, exhibits species- and tissue-specific expression patterns, suggesting a tightly controlled lipid supply system crucial for cardiac energetics under exercise or aging conditions [137]. UGP2, a key enzyme in UDP-glucose synthesis, may affect glycosylation processes and receptor function stability [138].

Despite lower FinalScore rankings, genes such as NRAP (force transmission [139]), SUCLA2 (protein succinylation [140]), DSP (intercellular adhesion [141]), and PDHA1 (glycolysis–TCA linkage [72]) may still contribute cooperatively to mitochondrial quality control, sarcomeric anchoring, and redox balance. DLD, a core component of α-ketoacid dehydrogenase complexes, induces mitochondrial stress responses and impairs neuromuscular function upon depletion, highlighting its potential importance in cardiac aging [142]. Although P2RX5 has been less studied in the cardiovascular system, as an ATP-gated ion channel, it may play a role in multisystem ionic homeostasis, thereby affecting cardiac adaptability and stress resistance [143]. TUBA4A regulates the cytoplasmic localization of YAP via microtubule sequestration and is involved in cardiac repair and regeneration following injury [144], while ACTA1 and SLC6A17, although not classically cardiac, may influence myocardial contractility or systemic adaptations through cytoskeletal or synaptic mechanisms [74,145].

The top-ranked gene based on the FinalScore was SMPX (Small Muscle Protein, X-linked), which exhibited high functional enrichment, network centrality, and mechanistic responsiveness. Specifically expressed in cardiac and skeletal muscle tissues, SMPX localizes to subsarcolemmal and sarcomeric junctions, where it stabilizes membrane structures under mechanical stress and participates in structural integrity, repair response, and adaptive signaling [146]. Its preferential expression in slow-twitch fibers suggests a specialized role in endurance-exercise-induced cardiac remodeling.

The critical status of SMPX (FinalScore = 1.307) is supported not only by its functional and network relevance but also by its potential regulatory mechanisms. SMPX is localized to submembranous domains in both cardiac and skeletal muscle [146,147], and chromatin accessibility in its distal X-chromosomal region is significantly elevated during aging [148]. The potential interacting protein of SMPX, Alpha Actinin 2 (ACTN2), is a critical component of the Z-disc, with its encoding gene playing a central role in sarcomere stability and mechanotransduction [149]. SMPX is primarily localized to subsarcolemmal regions and sarcomeric junctions, which serve as key interfaces for mechanical signal sensing and transduction in cardiomyocytes [147]. Previous studies have shown that SMPX may regulate the localization and structural integrity of ACTN2 through a membrane–cytoskeleton coupling mechanism, thereby preserving sarcomeric architecture and facilitating adaptive responses to mechanical loading [147,150]. This regulatory function is particularly relevant in mitigating age-related declines in myocardial contractile performance. The mechanosensitive role of SMPX has also garnered increasing attention in both clinical and experimental models of muscle disease. A multicenter study across several countries identified multiple missense mutations in SMPX associated with a novel form of distal myopathy. Affected individuals exhibited pathological features including subsarcolemmal vacuoles, sarcoplasmic inclusions, and fatty degeneration in muscle tissues. Cellular experiments further demonstrated that mutant SMPX proteins displayed enhanced aggregation and sequestration into stress granules, with impaired clearance, highlighting its importance in structural pathology and stress response [151]. In addition, studies have shown that mechanical stimuli such as stretch and shear stress can activate hemichannels, purinergic signaling, and mitochondrial reactive oxygen species (ROS) production, thereby remodeling calcium signaling and affecting cardiomyocyte contractility and electrophysiological stability [152]. As a subsarcolemmal stress sensor, SMPX may function upstream of this force–calcium–metabolism coupling axis to mediate mechanical signal perception and transmission.

Broader studies on Z-disc-mediated mechanoregulation further support the hypothesis of SMPX–ACTN2 coupling. In hypertrophic cardiomyopathy (HCM), myosin mutations that enhance cross-bridge formation activate mechanosensitive complexes centered around MLP (muscle LIM protein), leading to Z-disc destabilization, the disinhibition of the calcineurin–NFAT signaling pathway, and pathological hypertrophic remodeling [153]. Another study on truncating mutations in ACTN2 revealed disrupted interactions with membrane-associated proteins such as ACTN1 and GJA1, resulting in Z-disc–membrane uncoupling and abnormal calcium signaling [154]. These findings collectively reinforce the role of the Z-disc as a central hub for mechanical signal integration and suggest that SMPX regulation of ACTN2 is crucial for maintaining mechanostructural homeostasis. Notably, a recent study using a porcine model identified SMPX as a cooperating factor of MUSTN1 in promoting myogenic differentiation. MUSTN1 was shown to stabilize SMPX and preserve myofiber architecture, suggesting that SMPX possesses evolutionarily conserved functions in muscle development and biomechanical maintenance, despite the non-traditional nature of the animal model used [155].

Building upon this, it is important to note that the regulatory role of SMPX may not be limited to structural and mechanical responses but may also extend into metabolic and stress-adaptive domains, reflecting its multifaceted involvement in cardiomyocyte physiology. Missense mutations in SMPX increase its aggregation propensity and promote sequestration into stress granules, with impaired clearance observed in cultured cells [151]. While not directly linked to calcium signaling, stress granule formation is typically associated with oxidative stress [156,157]. Oxidative stress impairs mitochondrial dynamics, leading to mitochondrial fragmentation and excess ROS, which disrupt redox homeostasis—a core mechanism in cardiovascular pathology [158,159,160]. These findings suggest that SMPX may bridge energy metabolism and contractile function, providing a mechanistic framework for a metabolic–mechanical anti-aging axis induced by endurance exercise.

Intriguingly, a recent study by Hoelzl et al. (2025, *Nature Aging*) revealed that SMPX escapes X-chromosome inactivation (XCI) in cardiomyocytes, with escape levels further enhanced in aging mouse hearts [148]. This was associated with increased chromatin accessibility and structural openness in the distal X-chromosomal region [149]. The cell- and sex-specific regulation of SMPX expression implies potential roles in sex-biased cardiac aging and disease susceptibility.

From a translational perspective, the identification of SMPX and the proposed framework offer a conceptual basis for developing exercise mimetics [161,162,163]. Given the rising burden of aging and sedentary lifestyles [164,165,166,167], pharmacologically targeting SMPX or its interacting pathways may represent a feasible therapeutic alternative for individuals unable to engage in regular physical activity. Although molecular exercise surrogates are still in early development [161,162,163], this study provides a robust platform for target nomination and validation.

Despite its strengths in multi-omics integration and network modeling, this study has several limitations. First, the annotation data and interaction networks are derived from public databases, which may lack tissue- or cell-type specificity. Future studies should incorporate spatial transcriptomics or single-cell multi-omics to improve biological resolution. Second, the parameter settings in the FLAMES model (e.g., weight and penalty coefficients) were empirically defined; data-driven optimization using machine learning or Bayesian integration could enhance model adaptability. Lastly, while SMPX and other top candidates exhibit strong theoretical support, their functional roles require validation in vivo using knockout/overexpression models or organoid systems, particularly across sexes, age groups, and exercise intensities, to fully assess their feasibility as exercise therapeutic targets. However, the effector gene prioritization strategy established in this study identifies SMPX as a potential central node in the endurance-exercise-mediated attenuation of cardiac aging, offering a theoretical foundation and practical framework for elucidating the systemic mechanisms of exercise-induced anti-aging effects and informing future therapeutic development.

## 5. Conclusions

By integrating transcriptomic data from human cardiac aging with endurance-exercise-responsive datasets, 37 candidate effector genes were identified as key modulators of the exercise-mediated attenuation of cardiac aging. These genes act as molecular bridges connecting age-related functional decline with exercise-induced functional restoration. Enrichment analysis revealed that aging is primarily associated with mitochondrial dysfunction and sarcomeric disintegration, whereas endurance training promotes metabolic remodeling and structural adaptation. Core upstream kinases (such as TTN, EGFR, and the PDK family) and transcription factors (including NKX2-5, MYOG, and YBX3) were identified as central regulators of these processes. SMPX emerged as the top-ranked effector gene based on integrated prioritization, with demonstrated roles in mechano-metabolic coupling, redox balance, and sarcomere stability. Its ability to escape X-chromosome inactivation suggests additional relevance in the sex-specific trajectories of cardiac aging. Other high-priority genes, including KLHL31, MYPN, RYR2, SMYD1, and NDUFS1, contribute to a systems-level regulatory network governing calcium homeostasis, mitochondrial function, and structural integrity—key elements of the exercise-induced anti-aging response. While this study offers a theoretical foundation and prioritized targets for developing exercise-mimetic interventions, further in vivo functional validation is required.

## Data Availability

The data presented in this study are openly available in GeneSetCart at https://genesetcart.cfde.cloud/, https://doi.org/10.1093/gigascience/giaf025, reference: Marino, G.B., Olaiya, S., Evangelista, J.E., Clarke, D.J.B., Ma’ayan, A. GeneSetCart: assembling, augmenting, combining, visu-alizing, and analyzing gene sets. *Gigascience*. 6 January 2025; 14:giaf025. PMID: 40208796; PMCID: PMC11984350.

## Abbreviations

The following abbreviations are used in this manuscript:

ABL1	ABL Proto-Oncogene 1, Non-Receptor Tyrosine Kinase
ACAA2	Acetyl-CoA Acyltransferase 2
ACAT1	Acetyl-CoA Acetyltransferase 1
ACTA1	Actin Alpha 1, Skeletal Muscle
ACTN2	Alpha Actinin 2
AKT1	AKT Serine/Threonine Kinase 1
AMPK	AMP-Activated Protein Kinase
ARCHS4 Coexpression	All RNA-seq and ChIP-seq Sample and Signature Search Coexpression
ATP	Adenosine Triphosphate
BioGRID	Biological General Repository for Interaction Datasets
CAGE	Cap Analysis of Gene Expression
CAMK2A	Calcium/Calmodulin-Dependent Protein Kinase II Alpha
CASQ1	Calsequestrin 1
CAVIN4	Caveolae Associated Protein 4
CHCHD3	Coiled-Coil-Helix-Coiled-Coil-Helix Domain Containing 3
ChEA3	ChIP-X Enrichment Analysis 3
ChengKSIN	Cheng Kinase–Substrate Interaction Network
ChengPPI	Cheng Protein–Protein Interaction Dataset
CKMT2	Creatine Kinase, Mitochondrial 2
CVDs	Cardiovascular Diseases
dbGaP	Database of Genotypes and Phenotypes
DDN	Dendrin
DLD	Dihydrolipoamide Dehydrogenase
DNA	Deoxyribonucleic Acid
DSP	Desmoplakin
ENCODE ChIP-seq	ENCODE ChIP-Sequencing
Enrichr Queries	Enrichment Queries from Enrichr
FDR	False Discovery Rate
FGFR1	Fibroblast Growth Factor Receptor 1
FHL1	Four and a Half LIM Domains 1
FHL2	Four and a Half LIM Domains 2
FLAMES	Functional and Network Linear Model for Multi-dimensional Effector Scoring
GATA4	GATA Binding Protein 4
GO	Gene Ontology
GOT1	Glutamic-Oxaloacetic Transaminase 1
GSEA	Gene Set Enrichment Analysis
GTEx	Genotype-Tissue Expression
GTEx Coexpression	Gene Coexpression from GTEx Dataset
HIPPIE	Human Integrated Protein–Protein Interaction rEference
ID	Identifier
INSR	Insulin Receptor
JUN	Jun Proto-Oncogene, AP-1 Transcription Factor Subunit
KEA3	Kinase Enrichment Analysis 3
KLHL31	Kelch Like Family Member 31
Literature ChIP-seq	Literature-Curated Chromatin Immunoprecipitation Sequencing
LPL	Lipoprotein Lipase
LRRC2	Leucine Rich Repeat Containing 2
MAPK1	Mitogen-Activated Protein Kinase 1
mentha	MEtabolic Network Through Heterogeneous Analysis
MINT	Molecular INTeraction Database
MoTrPAC	Molecular Transducers of Physical Activity Consortium
MYF5	Myogenic Factor 5
MYF6	Myogenic Factor 6
MYL3	Myosin Light Chain 3
MYLK	Myosin Light Chain Kinase
MYOD1	Myogenic Differentiation 1
MYOG	Myogenin
MYPN	Myopalladin
NDRG4	N-Myc Downstream Regulated 4
NDUFS1	NADH:Ubiquinone Oxidoreductase Core Subunit S1
NFE2L1	Nuclear Factor, Erythroid 2 Like 1
NFIC	Nuclear Factor I C-Type
NKKX2-6	NK2 Homeobox 6
NKX2-5	NK2 Homeobox 5
NRAP	Nebulin Related Anchoring Protein
P2RX5	Purinergic Receptor P2X 5
PAXgene	PreAnalytix PaxGene Tissue/Tube System
PDHA1	Pyruvate Dehydrogenase E1 Alpha 1 Subunit
PDK1	Pyruvate Dehydrogenase Kinase Isozyme 1
PDK2	Pyruvate Dehydrogenase Kinase Isozyme 2
PDK3	Pyruvate Dehydrogenase Kinase Isozyme 3
PDK4	Pyruvate Dehydrogenase Kinase Isozyme 4
PDLIM5	PDZ and LIM Domain 5
PGC-1α	Peroxisome Proliferator-Activated Receptor Gamma Coactivator 1-Alpha
PhosDAll	Phosphorylation Data Aggregated from All Sources
PLN	Phospholamban
PPI	Protein–Protein Interaction
PPP1R3A	Protein Phosphatase 1 Regulatory Subunit 3A
prePPI	Predicted Protein–Protein Interactions Database
PRKCA	Protein Kinase C Alpha
PTMsigDB	Post-Translational Modification Signature Database
ReMap ChIP-seq	Regulatory Map ChIP-seq
RIN	RNA Integrity Number
RNA	Ribonucleic Acid
RPL3L	Ribosomal Protein L3-Like
RXRG	Retinoid X Receptor Gamma
RYR2	Ryanodine Receptor 2
SCN1A	Sodium Voltage-Gated Channel Alpha Subunit 1
SIRT1	Sirtuin 1
SIX1	SIX Homeobox 1
SLC6A17	Solute Carrier Family 6 Member 17
SMPX	Small Muscle Protein, X-Linked
SMYD1	SET and MYND Domain Containing 1
SOX18	SRY-Box Transcription Factor 18
STRING	Search Tool for the Retrieval of Interacting Genes/Proteins
STRING.bind	STRING Binding Evidence Subset
SUCLA2	Succinate-CoA Ligase ADP-Forming Beta Subunit
TBK1	TANK-Binding Kinase 1
TBX20	T-box Transcription Factor 20
TCA	Tricarboxylic Acid Cycle
TECRL	Trans-2,3-Enoyl-CoA Reductase Like
TF	Transcription Factor
TNNI3K	Troponin I-Interacting Kinase
ToppGene	ToppGene Suite Functional Enrichment Analysis Tool
ToppNet	ToppGene Suite Network Prioritization Tool
TRIM72	Tripartite Motif Containing 72
TTN	Titin
TUBA4A	Tubulin Alpha 4a
UGP2	UDP-Glucose Pyrophosphorylase 2
XIRP2	Xin Actin Binding Repeat Containing 2
YBX3	Y-box Binding Protein 3

## Appendix A

**Table A1:** Multi-dimensional prioritization scores and functional annotations of overlapping genes between endurance training and cardiac aging datasets.

Gene Symbol	Full Name	ToppGene Score	ToppNet Score	FinalScore	Primary Functional Annotation
ACAA2	Acetyl-CoA Acyltransferase 2	0.370033773	9.37 × 10^−5^	0.326818946	Fatty acid breakdown and mitochondrial lipid metabolism [135]
ACAT1	Acetyl-CoA Acetyltransferase 1	0.376042415	1.02 × 10^−4^	0.324654235	Cholesterol esterification and lipid metabolism [136]
ACTA1	Actin Alpha 1, Skeletal Muscle	0.565635952	3.07 × 10^−4^	0.000948507	Contractile cytoskeleton and myopathy/cardiomyopathy [74]
CASQ1	Calsequestrin 1	0.516803064	2.85 × 10^−6^	0.838524706	Calcium handling in fast-twitch muscle via sarcoplasmic reticulum [112]
CAVIN4	Caveolae Associated Protein 4	0.458419609	2.96 × 10^−6^	0.689971039	T-tubule development and function [118]
CKMT2	Creatine Kinase, Mitochondrial 2	0.556345232	4.38 × 10^−5^	0.812833829	Fast-twitch muscle calcium regulation [115]
DDN	Dendrin	0.254432411	4.23 × 10^−6^	0.171287172	Neural signaling and synaptic plasticity [130]
DLD	Dihydrolipoamide Dehydrogenase	0.468299535	2.36 × 10^−4^	0.167994144	Energy and pyruvate metabolism, redox balance [142]
DSP	Desmoplakin	0.401244892	1.82 × 10^−4^	0.22412071	Cell adhesion and myocardial stability [141]
FHL1	Four And A Half LIM Domains 1	0.413964729	9.28 × 10^−5^	0.406741081	Mechanical–transcriptional integration and muscle structure [123]
FHL2	Four And A Half LIM Domains 2	0.459343846	2.26 × 10^−4^	0.184490101	Muscle development, signaling and disease mechanisms [124]
GOT1	Glutamic-Oxaloacetic Transaminase 1	0.415075731	1.66 × 10^−4^	0.268865825	Amino acid metabolism and TCA anaplerosis [129]
KLHL31	Kelch Like Family Member 31	0.394484874	0	0.799148858	Sarcomere integrity and SR coupling [116]
LPL	Lipoprotein Lipase	0.377975045	1.69 × 10^−5^	0.463416222	Triglyceride hydrolysis and lipid metabolism [137]
LRRC2	Leucine Rich Repeat Containing 2	0.333212286	1.03 × 10^−6^	0.562245597	Mitochondrial function and cardiac remodeling [127]
MYL3	Myosin Light Chain 3	0.576717614	3.90 × 10^−5^	0.873185812	Sarcomere contraction and cardiac mechanics [112]
MYPN	Myopalladin	0.541188005	5.81 × 10^−5^	0.737031654	Sarcomere maintenance and signaling regulation [117]
NDRG4	NDRG Family Member 4	0.433742708	2.40 × 10^−5^	0.583950809	Stress response and cardiac maintenance [131]
NDUFS1	NADH:Ubiquinone Oxidoreductase Core Subunit S1	0.369426305	1.77 × 10^−4^	0.198506148	Mitochondrial metabolism, remodeling, and oxidative stress [84]
NRAP	Nebulin Related Anchoring Protein	0.466523641	1.29 × 10^−4^	0.415352267	Cardiac development and force transmission [139]
P2RX5	Purinergic Receptor P2X 5	0.218041283	1.22 × 10^−5^	0.077196996	ATP-dependent ion transport and multisystem regulation [143]
PDHA1	Pyruvate Dehydrogenase E1 Subunit Alpha 1	0.47683771	3.07 × 10^−4^	0	Glycolysis–TCA linkage and cardiac metabolic stability [72]
PDLIM5	PDZ And LIM Domain 5	0.481768861	1.23 × 10^−4^	0.452752432	Z-disc stability and signaling [122]
PLN	Phospholamban	0.498915554	5.74 × 10^−5^	0.650992399	Cardiac Ca^2+^ homeostasis and contraction [119]
PPP1R3A	Protein Phosphatase 1 Regulatory Subunit 3A	0.356625144	4.07 × 10^−5^	0.377933825	PP1 regulation, Ca^2+^ homeostasis, and rhythm control [133]
RPL3L	Ribosomal Protein L3 Like	0.422976684	1.94 × 10^−5^	0.567611129	Cardiac-specific translation elongation [126]
RYR2	Ryanodine Receptor 2	0.491528815	9.08 × 10^−5^	0.550565627	Ca^2+^ release and excitation–contraction coupling [120]
SCN1A	Sodium Voltage-Gated Channel Alpha Subunit 1	0.340497171	1.48 × 10^−5^	0.375326735	Action potential and neuro-cardiac rhythm [134]
SLC6A17	Solute Carrier Family 6 Member 17	0.186693123	2.72 × 10^−6^	0	Glutamine transport, synaptic function, and neurodevelopment [145]
SMPX	Small Muscle Protein, X-linked	0.527740575	1.55 × 10^−6^	1.307228059	Sarcolemmal stability and stress response [147,150,151]
SMYD1	SET And MYND Domain Containing 1	0.444368961	3.94 × 10^−5^	0.57604553	Cardiac development and epigenetic regulation [125]
SUCLA2	Succinate-CoA Ligase ADP-Forming Beta Subunit	0.452569875	1.32 × 10^−4^	0.387985714	TCA cycle catalysis and mitochondrial metabolism [140]
TECRL	Trans-2,3-Enoyl-CoA Reductase Like	0.268486979	1.16 × 10^−6^	0.313783778	Ca^2+^ homeostasis and rhythm stability [132]
TRIM72	Tripartite Motif Containing 72	0.317477311	2.07 × 10^−5^	0.312757183	Membrane repair and muscle cell integrity [128]
TUBA4A	Tubulin Alpha 4a	0.443812358	2.11 × 10^−4^	0.205730526	Microtubule structure and cardiac stress response [144]
UGP2	UDP-Glucose Pyrophosphorylase 2	0.261004361	3.85 × 10^−5^	0.166646811	Glucose metabolism and protein glycosylation [138]
XIRP2	Xin Actin Binding Repeat Containing 2	0.457391502	2.76 × 10^−5^	0.631808969	Actin structure and cardiac remodeling [121]

This table lists 37 genes identified at the intersection of cardiac aging (GTEx) and endurance exercise response (MoTrPAC). Genes are prioritized based on ToppGene functional similarity scores, ToppNet network centrality scores, and their linearly integrated FinalScore. Each gene entry includes its full English name and primary biological function. The FinalScore represents a composite prioritization metric obtained through FLAMES-based linear scaling of ToppGene and ToppNet scores, where higher values indicate greater potential centrality and regulatory importance within the cardiac aging–exercise adaptation network.
